# The Role of Glutathione Transferase Omega-Class Variant Alleles in Individual Susceptibility to Ovarian Cancer

**DOI:** 10.3390/ijms25094986

**Published:** 2024-05-03

**Authors:** Petar Simic, Vesna Coric, Igor Pljesa, Ana Savic-Radojevic, Nebojsa Zecevic, Jovana Kocic, Tatjana Simic, Vladimir Pazin, Marija Pljesa-Ercegovac

**Affiliations:** 1 Obstetrics and Gynecology Clinic Narodni Front, 11000 Belgrade, Serbia; simicp93@gmail.com (P.S.);; 2 Faculty of Medicine, University of Belgrade, 11000 Belgrade, Serbia; 3 Institute of Medical and Clinical Biochemistry, 11000 Belgrade, Serbia; 4 Center of Excellence for Redox Medicine, 11000 Belgrade, Serbia; 5 Gynecology and Obstetrics Centre Dr Dragiša Mišović, 11000 Belgrade, Serbia; 6 Serbian Academy of Sciences and Arts, 11000 Belgrade, Serbia

**Keywords:** ovarian cancer, glutathione transferase, *GSTO1*, *GSTO2*, haplotype, oxidative stress, ovarian neoplasms, risk factors, isoenzymes, female, adenocarcinoma

## Abstract

The tumor microenvironment is affected by reactive oxygen species and has been suggested to have an important role in ovarian cancer (OC) tumorigenesis. The role of glutathione transferases (GSTs) in the maintenance of redox balance is considered as an important contributing factor in cancer, including OC. Furthermore, GSTs are mostly encoded by highly polymorphic genes, which further highlights their potential role in OC, known to originate from accumulated genetic changes. Since the potential relevance of genetic variations in omega-class GSTs (*GSTO1* and *GSTO2*), with somewhat different activities such as thioltransferase and dehydroascorbate reductase activity, has not been clarified as yet in terms of susceptibility to OC, we aimed to investigate whether the presence of different *GSTO1* and *GSTO2* genetic variants, individually or combined, might represent determinants of risk for OC development. Genotyping was performed in 110 OC patients and 129 matched controls using a PCR-based assay for genotyping single nucleotide polymorphisms. The results of our study show that homozygous carriers of the *GSTO2* variant *G* allele are at an increased risk of OC development in comparison to the carriers of the referent genotype (OR1 = 2.16, 95% CI: 0.88–5.26, *p* = 0.08; OR2 = 2.49, 95% CI: 0.93–6.61, *p* = 0.06). Furthermore, individuals with *GST* omega haplotype H2, meaning the concomitant presence of the GSTO1*A and GSTO2*G alleles, are more susceptible to OC development, while carriers of the H4 (*A*A) haplotype exhibited lower risk of OC when crude and adjusted haplotype analysis was performed (OR1 = 0.29; 95% CI: 0.12–0.70; *p* = 0.007 and OR2 = 0.27; 95% CI: 0.11–0.67; *p* = 0.0054). Overall, our results suggest that *GSTO* locus variants may confer OC risk.

## 1. Introduction

Ovarian cancer (OC) represents the fifth most common cause of death in women, at the same time being the most lethal gynecological cancer [1]. The fundamental principles of ovarian cancer therapy have remained largely unchanged for over several decades. Since most patients seek the help of a gynecologist when their sickness is already in an advanced stage, surgical treatment continues to be considered an essential therapy [2]. In modern times, as the bulk of surgical cancer treatments are becoming less aggressive, ovarian cancer surgery is now performed exclusively by highly trained teams consisting of gynecologic oncologists and surgeons with various specialized backgrounds. Patients undergoing surgery in highly focused centers of excellence experience significantly improved overall and progression-free survival rates [3]. The objective of achieving complete eradication of residual disease can only be accomplished by the utilization of the most drastic surgical techniques.

Many years of work have gone into finding ways to detect ovarian cancer early because it is so hard to spot. Most common signs, like bloating and distention in the abdomen, pain in the lower abdomen, and frequent problems with the bladder and bowels, are very unpredictable and can be linked to a lot of different health conditions. Patients usually have to wait a while before they are identified because their first visit is usually to a gastroenterologist or general doctor, and then they are referred to a gynecologist [4,5].

Several tests have been made over the years that use ultrasound markers, a patient’s age, and their serum amounts of cancer antigen-125 (CA-125) to diagnose ovarian cancer. None of those tests were able to lower death rates or obtain good enough sensitivity and specificity to be used as a gold standard [6]. Now that artificial intelligence software is being built into ultrasound tools, there is hope that more will be done in the future for the early recognition of ovarian cancer.

One of the most important characteristics of ovarian cancer is its heterogeneity, from the molecular to the cellular and anatomical levels [7,8]. As a consequence, patients respond to surgery and/or systemic therapy with a high level of variability, while it also enables the development of chemoresistance, often leading to the development of aggressive, recurrent, and lethal cancer. Therefore, OC must neither be regarded nor treated as a single disease entity, since it comprises diverse subtypes with individual and complex molecular landscapes, which might even further alter as the tumor progresses [8,9]. What is more, the interactions between OC cells and the tumor microenvironment seem to further contribute to disease development and even affect their response to applied therapy. Studies conducted at the level of the genome, transcriptome, and proteome have even suggested the key role of the tumor microenvironment in ovarian cancer tumorigenesis [10].

The tumor microenvironment is highly affected by reactive oxygen species (ROS). Indeed, it is well established that cancer cells are under constant oxidative stress, to which they adjust using different mechanisms [11,12]. It is believed that ROS are responsible for the promotion of tumor initiation, tumor progression, and metastasis, but also participate in the development of drug resistance [11]. For that reason, the role of antioxidants has, on the one hand, been suggested in cancer prevention, but also in cancer treatment on the other [11]. The role of glutathione transferases (GSTs) in the maintenance of redox balance is considered as an important contributing factor in cancer [13,14]. These proteins are known for their various catalytic and noncatalytic functions, dominantly responsible for the biotransformation capacity towards xenobiotics, but also ROS [13,15,16]. What is more, GSTs participate in the regulation of signaling pathways involved in cell proliferation and cell death, suggesting their role not only in tumor development, but also in tumor progression [13]. It is important to note that GSTs are mostly encoded by highly polymorphic genes, which further highlights their potential role in OC, which is known to originate from accumulated genetic changes [16,17,18,19]. So far, cytosolic classes mu, theta, and pi have gained the most attention in OC, either as possible determinants of OC risk, prognostic factors, and/or modulators of drug resistance [19,20,21,22,23,24].

Omega-class glutathione transferases (*GSTO1* and *GSTO2*) represent a class with fairly different activities, including thioltransferase and dehydroascorbate reductase activity, as a consequence of cysteine presence in the active site [25,26]. *GSTO1* is known for its significant role in the glutathionylation cycle due to its deglutathionylase and glutathionylase activity, depending on diverse conditions [27]. Furthermore, by exhibiting deglutathionylase activity, *GSTO1* participates in the regulation of ryanodine receptors (type 1 ryanodine receptor, RyR1) as well as IL1-β activation [26]. Another suggested role of *GSTO1* refers to its regulation of signaling pathways involved in cell survival via the inhibition of pro-apoptotic MAPK signaling [28,29]. Interestingly, this protein has been found to be overexpressed in ovarian cancer [26]. Moreover, *GSTO1* has been shown to be involved in the conversion of protoporphyrin IX into heme in cells, in that way participating in the antitumor action of photodynamic therapy, while high levels of *GSTO1* expression have been found to be associated with the increased sensitivity to this type of treatment in different ovarian cancer cell lines [30]. Regarding *GSTO2*, its dehydroascorbate-reductase (DHAR) activity enables its role in the preservation of ascorbic acid in its reduced form, in that way supporting its role as an antioxidant which protects cellular components from free radical damage [31,32].

Although structurally somewhat different and exhibiting specific activities, omega-class GSTs share polymorphic expression with other GST classes. Polymorphisms recognized as the most important include two single nucleotide polymorphisms (SNPs): GSTO1*C419A (rs4925) and GSTO2*A424G (rs156697) [33,34,35]. The *GSTO1* rs4925 polymorphism, a consequence of alanine substitution with aspartate at position 140 (*Ala140Asp), leads to an alteration in enzyme deglutathionylase activity. For that reason, the GSTO1*C wild-type allele exhibits higher deglutathionylase and lower glutathionylase activity when compared to the GSTO1*A variant allele [27]. In the *GSTO2* rs156697 polymorphism, in which asparagine is substituted with aspartate at position 142 (*Asn142Asp), the association between the GSTO2*G variant allele and decreased *GSTO2* gene expression has been observed [36].

Since the potential relevance of *GSTO1* and *GSTO2* polymorphisms in the susceptibility to ovarian cancer has not been clarified as yet, we aimed to investigate whether the presence of different genetic variants of *GSTO1* and *GSTO2*, individually or combined, might represent determinants of risk for OC development.

## 2. Results

This case-controlled study comprised 129 controls and 110 patients who had previously been diagnosed with epithelial ovarian cancer. The patient group had a mean age of 58.14 years, while the control group was marginally younger at 57.05 years (*p* = 0.361). There were no statistically significant differences observed in terms of obesity, incidence of hypertension, or smoking between the two groups (*p* > 0.05). The average body mass index (BMI) of the patients was 25.79 ± 4.59, whereas it was 26.50 ± 4.71 in the control group (*p* = 0.253). In comparison to the control group (46%), the frequency of smoking was 53% in the patient group (*p* = 0.249). Table 1 details all available baseline demographic characteristics of patients diagnosed with ovarian cancer and the control group.

Regarding clinical characteristics, most patients had two births (55%), and 14 patients (13%) had a family history of ovarian or breast cancer. The majority of patients had high-grade tumors (89%), while only 11% of the patient group had low-grade tumors. When categorized by FIGO staging classification, 33 patients (30%) exhibited FIGO stage I, 25 patients (23%) FIGO stage II, 50 patients (46%) FIGO stage III, and the remaining 1% FIGO stage IV (Table 2).

The *GSTO1* rs4925 allele frequencies were as follows: GSTO1*C (count 331, proportion 0.69) and GSTO1*A (count 147, proportion 0.31). For *GSTO2* rs156697, they were as follows: GSTO2*A (count 319, proportion 0.67) and GSTO2*G (count 159, proportion 0.33). The data on the genotype distribution of *GSTO1* (rs4925) and *GSTO2* (rs156697) and the risk for the development of ovarian cancer in our study group are presented in Table 3. This particular analysis was computed in order to identify potential *GSTO* genotypes associated with an increased risk of ovarian cancer development as opposed to those *GSTO* genotypes that exhibited a protective effect. This was achieved by analyzing the individual effects of the GSTO1*C419A (rs4925) and GSTO2*A424G (rs156697) polymorphisms on the risk for the development of ovarian cancer by computing logistic regression analysis through a crude model (OR1), and afterwards confirming such findings through an adjusted model (OR2). 

As presented, the GSTO1*A allele and GSTO2*G allele were marked as those potentially associated with the modified risk of OC development [28,37]. However, although both *GSTO1* and *GSTO2* variant genotypes seem to increase the probability of developing OC, statistical analysis indicated the GSTO2*G allele as the ovarian-cancer-risk-associated one. More precisely, carriers of at least one GSTO2*G variant allele were at slightly increased risk, while homozygous carriers of variant *G allele exhibited more than 2-fold increased OC risk.

Through the non-random association of *GSTO* alleles, the influence of various *GSTO* haplotypes was calculated and expressed as the normalized coefficient of linkage disequilibrium (D’). Because the values for D’ are in a range from 0 to 1.0, the value 1.0 implies that two polymorphisms have the utmost association, while the 0 value implies that they are randomly associated.

**Figure 1:**
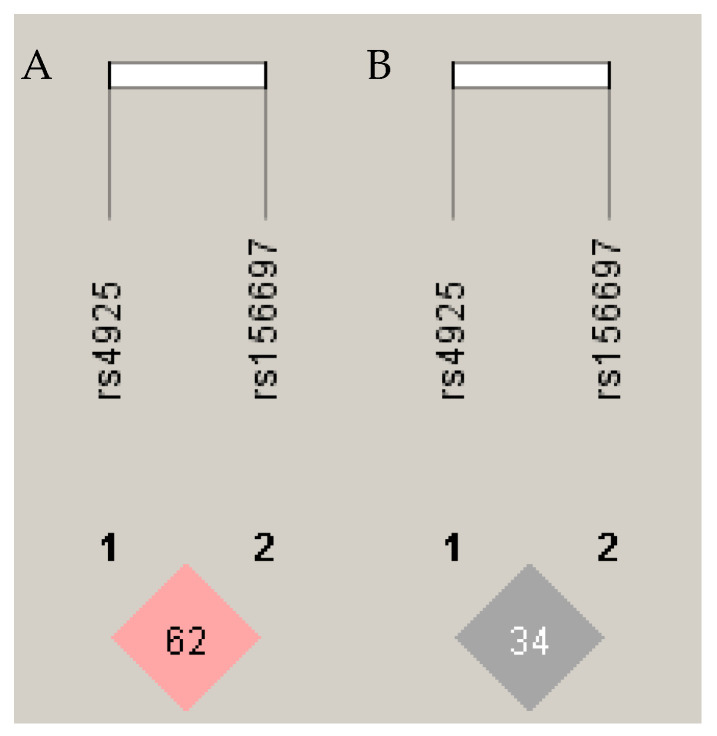
Non-random association of GSTO alleles, expressed as (**A**) the normalized coefficient of linkage disequilibrium (D’ = 0.62) and (**B**) a correlation coefficient (r^2^ = 0.34); D’ values can range from 0 to 1.0, with a value of 1.0 implying that two polymorphisms are associated in the most optimal manner, whilst a value of 0 shows that they are associated in a random manner. The picture was produced in the program Haploview (version 4.1).

We found a D’ of 0.62 between *GSTO1* rs4925 and *GSTO2* rs156697 (Figure 1A), confirming a moderately tight LD between these SNPs (*p* < 0.001). In addition, the correlation coefficient (r^2^) between the two loci was around 0.34 (Figure 1B).

Moreover, further analysis showed that the haplotype H4 (*A*A) exhibited lower risk of OC when crude and adjusted haplotype analysis was performed (OR1 = 0.29; 95% CI: 0.12–0.70; *p* = 0.007 and OR2 = 0.27; 95% CI: 0.11–0.67; *p* = 0.0054; Table 4). Indeed, the GSTO1*A allele and GSTO2*G allele, previously marked as those associated with an increased risk of OC development, exhibited the highest risk for OC development composing the haplotype H2 (OR1 = 1.47; 95% CI: 0.93–2.33; *p* = 0.10 and OR2 = 1.54; 95% CI: 0.93–2.56; *p* = 0.095).

**Figure 2:**
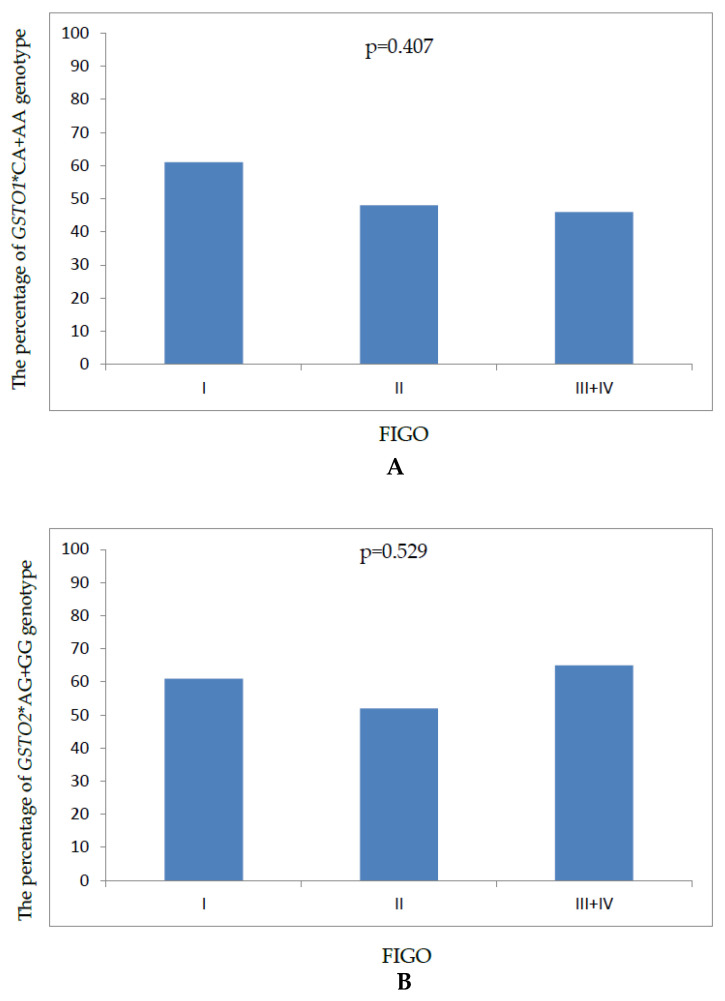
A possible genotype–phenotype association in ovarian cancer. (**A**). The percentage of GSTO1*CA + AA genotype carriers at different FIGO stages. (**B**). The percentage of GSTO2*AG + GG genotype carriers at different FIGO stages. FIGO, International Federation of Gynecology and Obstetrics staging classification.

In the next step, in order to assess a possible genotype–phenotype association, the percentage of GSTO1*CA + AA and GSTO2*AG + GG genotype carriers was determined for each FIGO stage. As presented in Figure 2, there was an insignificant decrease in the percentage of carriers of at least one GSTO1*A (Figure 2A) and a slight insignificant increase regarding the GSTO2*G (Figure 2B) variant allele, previously shown to affect OC risk, in relation to increasing FIGO stage.

## 3. Discussion

The results of our study have shown that homozygous carriers of the *GSTO2* variant G allele are at an increased risk of ovarian cancer development in comparison to carriers of the referent genotype. Furthermore, individuals with the *GST* omega haplotype H2, meaning the concomitant presence of the GSTO1*A and GSTO2*G alleles, are more susceptible to OC development, while carriers of the H4 haplotype (*A*A) exhibited a lower risk of OC when crude and adjusted haplotype analysis was performed. Keeping in mind the significant role of redox homeostasis in cancer development and progression, it seems reasonable to assume that certain SNPs that have been identified within genes encoding for antioxidant enzymes, such as superoxide dismutase, glutathione peroxidase, and glutathione transferase, might be deleterious to the antioxidant defense system [38]. This means that a particular antioxidant enzyme, which is the result of a gene mutation, is produced but fails to perform its function, or is produced but its action appears to be changed in a manner that is detrimental. In the case of GSTO isoenzymes, *GSTO1* plays a crucial part in protecting redox-sensitive protein thiol groups from irreparable oxidative damage, while *GSTO2* significantly contributes to the regulation of cellular redox balance [26,39]. Interestingly, more than thirty polymorphisms in the *GSTO1* gene and more than sixty polymorphisms in the *GSTO2* gene have been identified [36].

So far, an association between the GSTO1*C419A polymorphism (rs4925) and susceptibility to various cancers, including acute lymphoblastic leukemia, hepatocellular, breast, bile duct, non-small cell lung, colon, and testicular cancer has been confirmed [33,40]. On the other hand, the GSTO2*A424G polymorphism (rs156697) has been shown to be associated with ovarian, breast, urinary bladder, and renal cell cancers, which is in agreement with the results of our study [33,37,41,42,43]. The rationale for these associations might be both in the regulatory and antioxidant roles of omega-class glutathione transferases. Namely, the process of carcinogenesis is generally related to a disturbed redox balance in terms of a more reduced cellular state [12]. Among the multiple ways to regulate the function of various proteins involved in cancer development and progression, the process of the post-translational modification of proteins is recognized as a significant regulatory mechanism. More precisely, this type of regulation of protein activity is based on the simple principle of a molecular switch, causing tremendous changes to protein functions [44]. Apart from thoroughly investigated classical post-translational modifications of proteins, such as phosphorylation, acetylation, ubiquitination, and methylation, some novel types of modifications, including succinylation, hydroxybutyrylation, lactylation, and glutathionylation have emerged as a new manner of protein regulation [45]. Having in mind the recognized role of *GSTO1* in the process of glutathionylation and deglutathionylation, it seems reasonable to assume that presence of certain genetic variants of *GSTO1* might affect this process. Indeed, the *GSTO1* wild-type *C variant encodes the protein with the highest deglutathionylation activity [27]. Moreover, *GSTO1* was demonstrated to enhance the activation of the pro-inflammatory cytokine interleukin-1β (IL-1β) by post-translational processing [46].

Regarding *GSTO2*, the enzyme is known for its dehydroascorbate reductase activity which participates in the regeneration of dehydroascorbate [25]. Interestingly, it has been suggested that genetic variations consequential to the *GSTO2* rs156697 polymorphism affect *GSTO2* dehydroascorbate reductase [47]. In this manner, the presence of the GSTO2*G variant allele, especially in homozygous carriers, might lead to a significant decrease in enzyme dehydroascorbate reductase activity, resulting in decreased cellular ascorbic acid content. This might further significantly affect antioxidant capacity in homozygous individuals, potentially contributing to the process of carcinogenesis in susceptible individuals. Another process that might be affected by genetic variations in *GSTO2* is the hypoxia-inducible factor (HIF)-1 signaling pathway, suggested to be inhibited by ascorbic acid [31,48,49]. This transcriptional factor is responsible for the regulation of genes encoding proteins participating in energy metabolism, cell death, cell survival, and inflammation, all recognized as contributing factors in the process of carcinogenesis.

Taken together, the change in enzyme activity due to the presence of the mentioned deleterious SNPs, leading to the potential rise in the degree of oxidative stress, either on its own or in conjunction with other factors that have pro-oxidative potential, is likely to result in a particular milieu that contributes to the formation of tumors, such as ovarian cancer. Therefore, in this study, apart from the individual effect, an attempt was made to assess the haplotype effect of the *GSTO1* (rs4925) and *GSTO2* (rs156697) polymorphisms on the risk of OC development, since the haplotype effect of these *GSTO* polymorphisms is still controversial. This was accomplished by conducting two logistic regression risk models. We found that the carriers of the H2 haplotype (*A*G) were at a higher, yet not significant, risk of developing OC as opposed to the carriers of the H1 haplotype (*C*A). On the other hand, it seems that the H4 haplotype (*A*A) exerts a protective effect against OC development in comparison with carriers of the H1 (*C*A) haplotype.

Several studies have assessed GSTO haplotypes in cancer risk. The study of Petrovic et al. implies that carriers of the H7 haplotype (*GSTO1*rs4925*C, *GSTO2*rs156697*G, and *GSTO2*rs2297235*G) exhibited a 3-fold increased risk of testicular cancer compared to carriers of the H1 (*GSTO1*rs4925*C, *GSTO2*rs156697*A, and *GSTO2*rs2297235*A) haplotype; however, this did not reach statistical significance [40]. Interestingly, the very same haplotype exerted an increased risk of bladder cancer and clear renal cell carcinoma development [42,50]. However, the study of Djukic et al. assessed the *GSTO* haplotype comprising only *GSTO1*rs4925 and *GSTO2*rs156697, indicating that the *GSTO1*rs4925*C/*GSTO2*rs156697*G haplotype exhibited a higher odds ratio of bladder cancer development [41]. The findings of the preceding study do not totally agree with those of our investigation. The results of our study have indicated that the *GSTO1*rs4925 and *GSTO2*rs156697 H4 haplotype (*A*A) exhibited a significantly lower risk of OC when crude and adjusted haplotype analysis was performed (OR1 = 0.29 in model 1 and OR2 = 0.27 in model 2, *p* < 0.05). On the other hand, the GSTO1*A allele and GSTO2*G allele, previously marked as those associated with an increased risk of OC development, exhibited the highest odds ratios for OC within the H2 haplotype (OR1 = 1.47 in model 1 and OR2 = 1.54 in model 2, *p* > 0.05). The observed discrepancy in terms of differential haplotype effect on OC risk might be explained by the fact that omega-class GSTs exhibit regulatory roles in both inflammation and redox signaling; thus, the dual role of interplay between inflammation and oxidative stress might have both tumor-promoting and tumor-suppressing effects. Furthermore, the tumor microenvironment might also be affected by oxidative-stress-mediated carcinogenesis [51]. Undoubtedly, the *GSTO2*rs156697*G haplotype component seems to be associated with a higher risk of genitourinary malignancies. Similar findings were obtained by Pongstaporn et al., indicating that the presence of the GSTO2*G allele was associated with a 1.73-times higher risk of ovarian cancer development [37]. Having in mind that the GSTO2*A424G (rs156697) polymorphism may affect *GSTO2* protein levels, individuals carrying both variant GSTO2*G alleles may experience reduced *GSTO2* dehydroascorbate reductase activity, leading to impaired vitamin C recycling, disrupting redox balance [16,25,52]. The role of the association between vitamin C intake and metabolism with ovarian cancer risk regarding *GSTO* polymorphisms are yet to be deciphered.

However, a moderately tight linkage disequilibrium of assessed *GSTO* polymorphisms was obtained in this study. Linkage disequilibrium refers to the non-random association of alleles at two stated loci [53]. Though markers located on nonhomologous chromosomes are consistently segregated independently during meiosis, the same cannot be said for markers situated on the same chromosome. Indeed, on chromosome 10q, there is a region called a linkage locus that contains two *GST* genes of the omega class, *GSTO1* and *GSTO2* (rs4925 locus 104263031 and rs156697 with locus 104279427). These genes are separated by a distance of roughly 75 kilobases [54]. If the occurrence of allele *GSTO1* and the occurrence of allele *GSTO2* in a haplotype were separate events, then the two linked loci would have been said to be in linkage equilibrium (LE) [55]. On the other hand, *GSTO* alleles are said to be in linkage disequilibrium (LD) due to the fact that they do not occur randomly, but these alleles are found together more often than what would be predicted based on the natural recombination of DNA [56]. Consequently, the allele of one polymorphism in an LD block (haplotype) can predict the allele of the adjacent polymorphism. The underlying mechanism comprises the process of recombination, commonly known as crossing over [55].

In summary, our results suggest that *GSTO* locus variants may confer OC risk. Preferably, *GSTO2* should be primarily sequenced for variants that may influence disease risk. Assessing the connection of *GSTO* polymorphisms with expression levels in the future could offer a distinct opportunity to pinpoint the specific disease gene at the linkage or association locus.

It is important to discuss a few of this study’s limitations. Though the case-controlled study design is thought to be a useful method for identifying risk biomarkers, selection bias could undermine the accuracy of the study findings. Apart from data on smoking, our analysis did not utilize the data on occupational or environmental exposure due to their unavailability. Furthermore, the patients’ positive family history of ovarian or breast cancer was confirmed solely based on the anamnestic data, while, unfortunately, neither BRCA mutation nor other homologous recombination deficiency status was determined. Moreover, the haplotype analysis did not include family data on assessed polymorphisms. Finally, the lack of significant results when individual *GSTO* genotypes were assessed can be explained by the rather small sample size.

## 4. Materials and Methods

This case-controlled study was conducted in compliance with the principles outlined in the Helsinki Declaration, as well as with the approval of the Ethics Board of the Faculty of Medicine, University of Belgrade, Serbia, and the Ethics Board of the University Gynecology and Obstetrics Clinic “Narodni front”, Belgrade, Serbia. Written informed consent was obtained from all recruited subjects.

This case-controlled study included 110 women who were newly diagnosed with epithelial ovarian cancer and 129 subjects comprising the control group. These subjects were age-matched and did not have any previous personal history of cancerous disease. The control DNA samples, as well as part of the OC patient samples, are a part of the DNA biobank located at the Institute of Medical and Clinical Biochemistry, Faculty of Medicine, University of Belgrade. The World Health Organization’s criteria for classifying tumors of female reproductive organs were followed in the diagnosis of ovarian cancer (serous, endometrioid, mucinous, and clear cell), and the International Federation of Gynecology and Obstetrics (FIGO) staging system was used for staging.

All information, including the grade and stage of differentiation of the tumors, was extracted from the pathological and surgical reports contained in the patients’ records. Tumor grade was determined based on architectural pattern and mitosis index. Women who had a prior diagnosis of ovarian cancer or any other neoplasm were excluded from participation. Interviews were conducted to collect information on the subjects’ general health, gynecological and medical history with a focus on reproductive life, and previous and/or current malignant diseases. A structured questionnaire was used to gather lifestyle details, including occupation and professional history, cigarette smoking habits, and alcohol consumption. The patients’ smoking habits regarding cigarettes were documented and subsequently classified into two groups: those who never smoked and those who did so at some point.

Through the utilization of a commercial kit (The PureLinkTM Gel Extraction Kit #K210025, manufactured by Invitrogen in Waltham, MA, USA), genomic DNA was extracted from whole blood samples that were acquired at the time of diagnosis. TaqMan SNP Genotyping assays (Life Technologies, Applied Biosystems, Carlsbad, CA, USA) were applied for analyzing the following genotypes of GSTO1*C419A (rs4925) (ID assay number: C_11309430_30) and GSTO2*A424G (rs156697) (ID assay number: C_3223136_1), in accordance with the instructions provided by the manufacturer.

SPSS, version 17 (SPSS Inc., Chicago, IL, USA) was employed for the following statistical assessment: the χ2 test (for the differences between categorical variables and the genotype distribution with respect to the Hardy–Weinberg equilibrium), the independent samples *t*-test for continuous variables, and logistic regression analysis (for the association between the *GSTO* genetic variations and the risk for the development of ovarian cancer). The latter was accomplished by calculating odds ratios (ORs) and confidence intervals (CIs) with a 95% success rate. Two risk models were applied: model 1 was derived without any adjustments (crude OR), and model 2 was derived with adjustments to age, hypertension, smoking, and body mass index (BMI). To determine the degree of linkage disequilibrium (LD) that exists between pairs of SNPs, the software program Haploview (version 4.1, Broad Institute, MIT, Harvard, MA, USA) was utilized. Additional utilization of SNPStats allowed for the confirmation of the impact that various *GSTO* haplotypes had on the odds of developing ovarian cancer. A *p*-value that was less than or equal to 0.05 was regarded as statistically significant.

## 5. Conclusions

The fact that certain genetic variants of antioxidant enzymes—specifically, omega-class glutathione transferases—independently or combined affect individual predisposition to ovarian cancer development further emphasizes the involvement of genetic susceptibility in this complex disease. However, further studies are necessary in order to elucidate the exact roles of specific enzymes, as well as their possible therapeutic potential, if any.

## Figures and Tables

**Table 1:** Available baseline demographic characteristics of patients with ovarian cancer and controls.

Parameters ^1^	Patients, n (%)	Control, n (%)	*p*-Value
Age (years)	58.14 ± 9.8 ^2^	57.05 ± 8.10	0.361
Obesity			
BMI < 25	53 (49)	48 (42)	0.297
BMI > 25	55 (51)	66 (58)	
BMI (kg/m^2^)	25.79 ± 4.59 ^2^	26.50 ± 4.71	0.253
Smoking ^3^			
Never	50 (47)	70 (54)	0.249
Ever	57 (53)	59 (46)	
Hypertension			
Yes	34 (32)	49 (38)	0.296
No	74 (68)	80 (62)	

^1^ Available data; ^2^ presented as mean ± SD; ^3^ at least 60 cigarettes smoked prior to study onset; BMI: body mass index.

**Table 2:** Clinical characteristics of patients with ovarian cancer.

Parameters ^1^	Patients, n (%)
Parity	
0	12 (11)
1	27 (25)
2	60 (55)
>3	11 (9)
Family history of ovarian cancer	
Yes	14 (13)
No	96 (87)
FIGO stage	
I	33 (30)
II	25 (23)
III	50 (46)
IV	2 (1)
Grade of tumor	
I	11 (11)
II	55 (52)
III	39 (37)

^1^ Available data; FIGO, International Federation of Gynecology and Obstetrics staging classification.

**Table 3:** GST genotypes in relation to the risk of ovarian cancer.

*GST* Genotype	Patients n (%)	Controls n (%)	OR1 (95% CI)	*p* Value	OR2 (95% CI)	*p* Value
*GSTO1*						
*CC	54 (49)	59 (46)	1.00 *		1.00 *	
*CA	42 (38)	63 (49)	0.72 (0.42–1.24)	0.25	0.72 (0.40–1.27)	0.25
*AA	14 (13)	7 (5)	2.18 (0.82–5.81)	0.11	2.09 (0.72–6.05)	0.17
*CC	54 (49)	59 (46)	1.00 *		1.00 *	
*CA-AA	56 (51)	70 (54)	0.87 (0.52–1.45)	0.60	0.84 (0.49–1.46)	0.55
*GSTO2*						
*AA	43 (39)	62 (48)	1.00 *		1.00 *	
*AG	52 (47)	57 (44)	1.31 (0.76–2.25)	0.32	1.43 (0.80–2.55)	0.21
*GG	15 (14)	10 (8)	2.16 (0.88–5.26)	0.08	2.49 (0.93–6.61)	0.06
*AA	43 (39)	62 (48)	1.00 *		1.00 *	
*AG-GG	67 (61)	67 (52)	1.44 (0.86–2.41)	0.16	1.57 (0.90–2.73)	0.10

* Reference group; OR1, crude odds ratio; OR2, adjusted to age, hypertension, smoking, and body mass index (BMI); CI, confidence interval.

**Table 4:** The haplotype effect of *GSTO* genotypes on the risk of ovarian cancer development.

*GSTO1*	*GSTO2*	Count (Frequency)	OR1 (95% CI)	*p*-Value	OR2 (95% CI)	*p*-Value
*C*	*A*	129 (0.58)	1.00 *		1.00 *	
*A*	*G*	51 (0.22)	1.47 (0.93–2.33)	0.10	1.54 (0.93–2.56)	0.095
*C*	*G*	23 (0.10)	0.76 (0.39–1.48)	0.57	0.82 (0.41–1.63)	0.57
*A*	*A*	18 (0.08)	0.29 (0.12–0.70)	0.007	0.27 (0.11–0.67)	0.005

* Reference group; OR1, crude odds ratio; OR2, adjusted to age, hypertension, smoking, and body mass index (BMI); CI, confidence interval.

## Data Availability

The data supporting reported results can be found upon request in the form of datasets available at the Institute of Medical and Clinical Biochemistry, Faculty of Medicine, University of Belgrade.
